# A Novel Epidermis Model Using Primary Hidradenitis Suppurativa Keratinocytes

**DOI:** 10.1155/2024/4363876

**Published:** 2024-02-27

**Authors:** Isabel Haferland, Andreas Pinter, Tanja Rossmanith, Sandra Diehl, Claudia Buerger, Tanja Ickelsheimer, Roland Kaufmann, Anke Koenig

**Affiliations:** ^1^Goethe University Frankfurt, University Hospital, Department of Dermatology, Venereology and Allergology, Theodor-Stern-Kai 7, Frankfurt am Main 60590, Germany; ^2^Fraunhofer Institute for Translational Medicine and Pharmacology ITMP, Theodor-Stern-Kai 7, Frankfurt am Main 60596, Germany

## Abstract

Hidradenitis suppurativa (HS) is a chronic inflammatory skin disease. Patients can present with inflammatory nodules, abscesses up to fistulas, or sinus tracts in intertriginous body parts. Occlusion of the sebaceous gland unit leads to its rupture, with a subsequent exuberant immune response. Given there is still no causative therapy, to better understand HS and develop novel therapeutic concepts, research activities in the HS field are constantly growing. Primary skin cells, blood cells, and *ex vivo* explant cultures from HS patients have been previously used as HS cell culture models. *In vitro* reconstituted epidermal models are established to study inflammatory dermatoses, such as psoriasis or atopic dermatitis. For HS, the exploration of epidermis models would be an excellent addition, e.g., biomarkers or barrier function in testing new topic treatment options. We therefore established a stratified *in vitro* HS epidermis model based on primary cells from HS lesions. After isolating keratinocytes from lesional skin, we cultured them submerged in a transwell system. To induce differentiation, we then lifted them to the air-liquid interface. Immunohistochemical staining demonstrated that our HS-epidermis model meets the expected differentiation pattern. In addition, we detected the secretion of the inflammatory cytokines interleukin-1*β* and TNF-*α*.

## 1. Introduction

Hidradenitis suppurativa (HS) is a chronic inflammatory skin disease with a prevalence range of 0.04–4% [1, 2]. Clinical symptoms include painful nodules, abscesses to fistula tracts, and sinus tract formation [3, 4]. Initial histological events are considered to include hyperkeratosis and perifolliculitis in the sebaceous gland unit, resulting in occlusion, inflammation, and rupture of the hair follicle [5–7]. Subsequently, released bacteria and damage-associated molecular patterns and keratins induce an aberrant immune reaction [8].

As the exact pathophysiology of this autoinflammatory disease is not yet fully understood, there are no HS causative therapies, making HS one of the most studied skin diseases. *In vitro* approaches mostly use primary cells from the blood or skin of HS patients [9–12]. HS typically starts after puberty and reaches its peak in the third and fourth decades of life [13–15]; thus, the primary skin cells of HS are adult cells. It is important to note that adult cells have lower proliferation rates when compared to juvenile cells [16]. As an alternative to using primary cells, cell lines or healthy primary cells are treated with various cytokines associated with HS to simulate an inflammatory environment [9, 12, 17]. *In vitro* research demonstrated that an impaired Notch signaling pathway can cause a disorder in the differentiation of keratinocytes [18]. Furthermore, keratinocytes found in the affected areas of HS skin produce various chemokines and cytokines that serve to attract additional immune cells [19, 20]. For *ex vivo* studies, punch biopsies, so-called explants, are taken from lesional and nonlesional HS tissues and cultured in a transwell system with the epidermis exposed at the air-liquid interface. This system presents an ideal setup for basic research as well as drug and biomarker screening in the laboratory, as nearly all the characteristics of the native tissue are represented [9, 21–30]. Still debated is whether established animal models for other diseases, such as acne-like diseases, are also suitable as animal models for HS. These models include canine HS, German Shepherd dog pyoderma, *γ*-secretase knockout mice, and aryl hydrocarbon receptor-mediated chloracne [31]. Recently, Quartey et al. established an HS xenograft mouse model by transplanting lesional HS tissue onto immunodeficient NOD-scif gamma mice expressing human IL-3, granulocyte/macrophage colony-forming factor and stem cell factor [32]. Another mouse model, presented by Yang et al., exhibits the main phenotypic characteristics of HS, such as hyperkeratosis of the hair follicle and inflammation through keratin 5-Cre-driven epidermis-specific nicastrin conditional knockout [33].

An *in vitro* model that has been widely used in other inflammatory skin diseases, such as atopic dermatitis and psoriasis, utilises reconstructed human skin equivalents. Among reconstructed skin models, a distinction needs to be made between full-thickness skin and epidermis equivalents [34–36]. Reconstructed human skin equivalents are currently used in basic research and in cosmetics and drug testing, as an alternative to animal testing [37–42]. To our knowledge, a skin equivalent has not yet been developed for HS. This would also be a good addition to the models established for the investigation of biomarkers, or topical and systemic drug testing, as well as for basic research. Although HS inflammation is deep-seated, epidermis models remain crucial. A study by Schell et al. 2023 reconfirmed the significance of keratinocytes in HS inflammation [19]. When working with primary cells, it is important to focus on the epidermal layer, as it is a crucial part of the skin model. Therefore, it is recommended to start by working on the epidermal layer. Epidermis models are highly suitable for studying barrier function. Similar disturbances have already been observed for psoriasis and been published [43]. Although *ex vivo* explants are currently the best model for HS research, this model has limitations. The biopsies must be placed in culture immediately after eviscerating them from the tissue. By isolating primary cells from the tissue for skin models, experiments can be more flexibly integrated into daily research routines. Skin models can be cultivated without the need for additional biopsies. Here, tissue that is typically discarded during surgical excisions can be used. Furthermore, epidermis models provide other advantages with regards to genetic manipulations or can serve as a substitute for animal models, an approach already being implemented in the cosmetics industry [44–46].

Therefore, we aimed to examine whether primary keratinocytes derived from lesional HS skin can form HS-like epidermal models. In this context, we determined the expression of specific epidermal differentiation markers in lesional HS skin compared to HS-epidermis equivalents and their corresponding controls. Additionally, we assessed the cytokine production of the HS epidermis models to evaluate whether they are suitable as models for HS research.

## 2. Materials and Methods

### 2.1. Patient Collective and Ethics

Heterogeneous lesional skin was obtained from excised skin after radical surgery of clinically confirmed diagnoses of HS Hurley II or Hurley III at the Department of Dermatology, Venerology and Allergology of the University Hospital in Frankfurt am Main, Germany, from September 2018 to January 2021. Infantile foreskin used as control was obtained after clinical routine surgery (circumcision) at the Center of Pediatric and Adolescent Medicine of the University Hospital in Frankfurt am Main, Germany, from September 2018 to November 2019. Breast skin (mama reduction plastic) used as control was obtained after mammary reduction at the Department for Gynecology and Obstetrics of the University Hospital in Frankfurt am Main, Germany, from May 2019 to August 2020. All works accomplished on these tissues were carried out in accordance with the Declaration of Helsinki and in agreement of the ethics committee of the University Hospital Frankfurt am Main (Local Ethics Commission/institutional review board). The surgical material was delivered anonymously; therefore no patient-related data were collected. Six-millimeter punch biopsies were taken from actively inflamed HS lesions from the surgically removed material, as well as mammary skin as control, and fixed in 4% formalin.

### 2.2. Cell Culture

Keratinocytes of lesional skin of HS and infantile foreskin were isolated after enzymatic digestion with thermolysin (Sigma Aldrich). Cells were cultured in Keratinocyte Growth Medium-2 (Promocell) with 1% penicillin-streptomycin solution until the first medium replacement.

### 2.3. Cultivation of Epidermis Models

Primary keratinocytes from HS patients or healthy foreskin were seeded on ThinCert™ cell culture inserts (Greiner bio-one), as previously described [37]. After a submerse cultivation period in CnT-PRIME medium (CellnTec, Bern), the medium was replaced by CnT-PR-3D medium (CellnTec) every two days and lifted to air-liquid interface to induce epidermal differentiation. Epidermis models were cultured at 37°C and 5% CO_2_ in a humidified incubator. Medium was collected on days 14 and 21 of the air-liquid interface and frozen at −20°C until further investigation. After the culturing period, the models on the membranes were cut from the insert and fixed in 4% formalin.

### 2.4. Immunohistological Staining

Epidermal Models and biopsies were embedded in paraffin, sectioned at 4 *µ*m and stained with hematoxylin (Merck) and eosin (Microm International). Immunohistological staining was performed using the following primary antibodies: anti-cytokeratin-10 (abcam), anti-filaggrin (Santa Cruz), anti-involucrin (abcam) and anti-Ki-67 (DCS). For negative control, the same epidermal model was stained with an isotype antibody of irrelevant specificity. Immunostaining was visualized using the alkaline phosphatase method and by hematoxylin nucleus staining. Images were taken with a Nikon eclipse Ni microscope at 10-fold magnification. Quantification was obtained using the ImageJ software (version 1.53q, Wayne Raband, National Institutes of Health, USA). Epidermal thickness was quantified by measuring epidermal length from *stratum basale* up to and including the *stratum granulosum* at five locations and then calculating the mean value. To examine the intensity of cytokeratin-10, filaggrin and involucrin staining within the epidermis, the mean gray values of the epidermis were measured within a defined area. Hematoxylin-stained cell nuclei were separated by color deconvolution. A threshold was set to ensure that only the stained area was used for the measurement. Ki-67 stained cells were visualized and quantified by normalizing these to the total counted number of nuclei in the epidermis.

### 2.5. Enzyme-Linked Immunosorbent Assay

To assess the inflammatory profile of the HS epidermis models, IL-1*β* and TNF-*α* were measured in the culture medium after 14 or 21 days of culture using enzyme-linked immunosorbent assay (R&D systems). The assays were used according to the manufacturer's instructions. Standard curve was created by generating a four-parameter logistic curve fit.

### 2.6. Statistical Analysis

For statistical calculation, GraphPad Prism version 5 was used. The Wilcoxon Mann Whitney *U* Test was performed to evaluate statistical significance by using two-tailed test. This test was selected because the data did not follow a normal distribution, but the samples were independent. The figure legends indicate the number of replicates. *p* value ≤ 0.05 was considered statistically significant and was indicated within figures as ^*∗*^*p* < 0.05, ^*∗∗*^*p* < 0.01 and ^*∗∗∗*^*p* < 0.001.

## 3. Results

**Figure 1:**
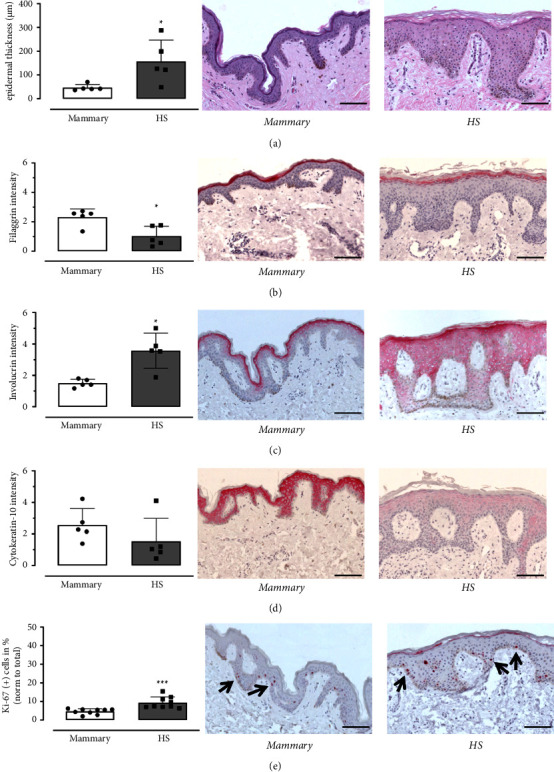
Staining of non-inflammatory control (mammary) and hidradenitis suppurativa lesional (HS) biopsies. The biopsies were stained with (a) hematoxylin-eosin as well as (b) anti-filaggrin, (c) anti-involucrin, (d) anti-cytokeratin-10, and (e) anti-Ki-67 antibodies. Representative sections are shown and the value were mean ± SD (hematoxylin-eosin *n* = 5; filaggrin *n* = 5; involucrin *n* = 5; cytokeratin-10 *n* = 5; Ki-67 *n* = 9 patients; scale bar = 100 *µ*M; black squares = mammary; black dots = HS; and black arrows = Ki-67(+) cells). *p* values were calculated using Wilcoxon Mann–Whitney *U* Test, *p*^*∗*^ < 0.05, *p*^*∗∗*^ < 0.01, *p*^*∗∗∗*^ < 0.001.

The adult cells from HS lesions used in the epidermis models formed a multi-layered epidermis. To validate the staining of cytokeratin-10, filaggrin, involucrin and Ki-67 expression, these markers of differentiation were determined within the epidermis of both the mammary control and HS donors. Hematoxylin-eosin staining shows significantly increased epidermal thickness with increased and irregular rete ridges in HS, compared to mammary skin (*p* value 0.0159). Staining of filaggrin is reduced in HS compared to mammary skin (*p* value 0.0317). While the distribution of involucrin in mammary skin is decreased and limited to the *stratum granulosum*, it extends to the *stratum spinosum* in HS (*p* value 0.0119). Immunohistological detection of cytokeratin-10 indicates decreased expression in HS compared to mammary skin, with no differences in the distribution of cytokeratin-10 between HS and mammary skin (*p* value 0.0952). Quantification of proliferating keratinocytes in the epidermis shows an increased number of Ki-67(+) keratinocytes in HS in comparison with mammary skin (*p* value < 0.0001). In contrast to mammary skin, Ki-67(+) keratinocytes are also located in the *stratum spinosum* and *stratum granulosum* in HS epidermis (Figure 1).

**Figure 2:**
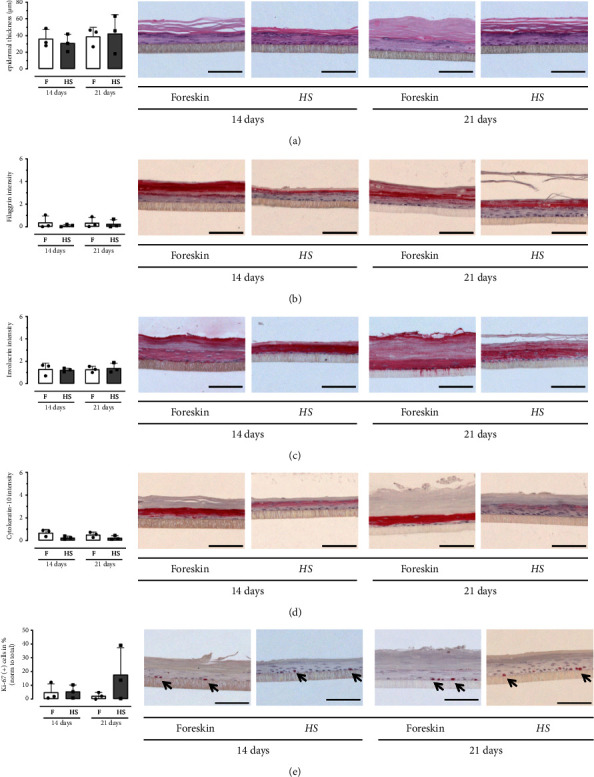
Staining of the epidermis models of foreskin (f) and hidradenitis suppurativa (HS) keratinocytes. Epidermis models were cultivated for 14 or 21 days at the air-liquid interface. The epidermis equivalents were stained with (a) hematoxylin-eosin as well as (b) anti-filaggrin, (c) anti-involucrin, (d) anti-cytokeratin-10, and (e) anti-Ki-67 antibodies. Representative sections are shown and the values were mean ± SD (*n* = 3; scale bar = 100 *µ*M; black squares = foreskin; black dots = HS; and black arrows = Ki-67(+) cells). *p* values were calculated using Wilcoxon Mann–Whitney *U* Test, which showed no statistically significant differences between both groups.

We cultured the epidermis models from HS keratinocytes and healthy control keratinocytes from foreskin for 14 and 21 days as described in Borowczyk et al. [37] and stained sections of the models with hematoxylin-eosin. Keratinocytes from both HS tissue and foreskin present multiple and stratified cell layers. When comparing the morphology of the models, there is no difference in epidermal thickness between duration of cultivation (14 days *p* value 0.7000, 21 days *p* value 1.000), whereas the thickness of the *stratum corneum* seems to be increased with prolonged cultivation. Moreover, the differentiation markers were stained immunohistochemically to evaluate if the functional layers of the epidermis were formed by the HS keratinocytes in the epidermis equivalents. The intensity of filaggrin expression suggests a slight decrease in the HS-epidermis models compared to the foreskin-epidermis equivalents after 14 days of cultivation (14 days *p* value 1.000), it then remains in the same proportion after 21 days of cultivation (21 days *p* value 0.7000). Immunohistochemistry of involucrin indicates more intense staining in HS-epidermis equivalents compared to foreskin-epidermis models (14 days *p* value 0.7000). This effect is also seen with prolonged cultivation (21 days *p* value 1.000). Expression of cytokeratin-10 shows lower intensity in HS than in foreskin-epidermis models after 14 days of cultivation (14 days *p* value 0.2000) and remains unchanged after prolonged cultivation (21 days *p* value 0.2000). The foreskin-epidermis equivalents exhibit keratinocytes with less proliferative capability, as indicated by Ki-67 positive cells compared to HS-epidermis models (14 days *p* value 1.000, 21 days *p* value 0.4000, Figure 2).

**Figure 3:**
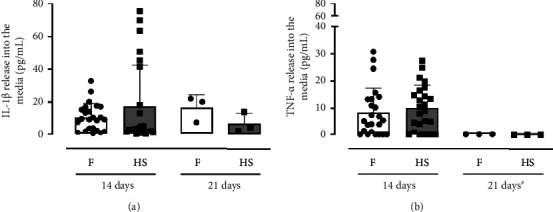
Inflammatory profile of the epidermis models of foreskin (F) and hidradenitis suppurativa (HS) keratinocytes measured by (a) interleukin-1*β* (IL-1*β*) and (b) tumor necrosis factor alpha (TNF-*α*) enzyme-linked immunosorbent assay. The values were mean ± SD 14 days = *n* = 24, 21 days *n* = 3; black squares = foreskin; black dots = HS; and ^#^ = below detection limit. *p* values were calculated using Wilcoxon Mann–Whitney *U* test, which showed no statistically significant differences between both groups.

To investigate whether epidermis models derived from HS keratinocytes secrete the pro-inflammatory cytokines IL-1*β* and TNF-*α*, we examined the release of IL-1*β* and TNF-*α* into the medium of the epidermis equivalents. A non-significant increased secretion of IL-1*β* by the HS-epidermis models compared to the foreskin-epidermis models after 14 days of cultivation could be detected (14 days *p* value 0.4455). When culturing for a total of 21 days, the trend of increased secretion is noted in the foreskin-epidermis models (21 days *p* value 0.2000, Figure 3(a)). The level of secreted TNF-*α* into the medium after 14 days of cultivation suggests a non-significant tendency for higher secretion by the HS-epidermis models (14 days *p* value 0.3303). After a cultivation period of 21 days, there is no TNF-*α* measurable in the medium of the HS- and foreskin-epidermis models (Figure 3(b)).

## 4. Discussion

To date, an epidermal *in vitro* model, as established for other inflammatory dermatoses [34–36], has not been developed for HS. Therefore, our study investigated whether keratinocytes derived from lesional HS skin form a multi-layered stratified epidermis and recapitulate features of HS skin, compared to models from healthy keratinocytes. Since the disease usually reaches its peak of activity in the third and fourth decades of patients' lives [13–15], the cells used to generate the HS epidermis models were adult keratinocytes. Adult keratinocytes are considered less proliferative than juvenile keratinocytes [16]. The foreskins used as a source of healthy keratinocytes for the epidermal models were from infant donors. To prevent the proliferation limitations of adult keratinocytes, we first attempted different cultivation protocols (data not shown). During this process, we demonstrated for the first time that lesional HS keratinocytes form multi-layered epidermis models *in vitro*. Several studies show that the HS epidermis displays increased rete ridges and psoriasiform hyperplasia in HS in comparison to non-inflammatory skin [7, 47–49]. We saw this typical tissue architecture in immunehistochemistry stainings of HS biopsies, but unfortunately could not reproduce it in our *in vitro *HS-epidermis models. The absence of rete ridges in the epidermis models is not surprising, given the formation of rete ridges is only expected in a full-thickness skin model. However, after 21 days of cultivation, we observed a thickened *stratum corneum* when compared to 14 days of cultivation. In line with the fact that the epidermis is permanently self-regenerating, the thickened *stratum corneum* is probably due to a prolonged cultivation period and thus more time enabled the development of the *stratum corneum*. Both HS biopsies and HS-epidermis models showed reduced filaggrin intensity regardless of the cultivation time of the epidermis equivalents. Filaggrin is an important component of the cornification process and as such, is an essential contributor to the stratification of the epidermis [49]. Dmitriev et al. recently showed decreased filaggrin expression in lesional HS skin compared to healthy controls [43, 49], which is consistent with our findings. In addition, this effect is reported in other inflammatory skin diseases such as atopic dermatitis or psoriasis [43, 50, 51]. Loss-of-function mutation of filaggrin has been described in atopic dermatitis and is associated with a decreased skin barrier function [52, 53]. Another protein involved in the initial steps of the cornification process is involucrin [54]. We observed an altered involucrin distribution in the biopsies of HS skin compared to non-inflammatory control biopsies. This effect was also reflected in the immunohistological staining of our HS-epidermis models. Compared to healthy controls, Dmitriev et al. also saw increased involucrin expression in lesional HS as well as lesional psoriasis skin [43]. The elevated involucrin expression has similarly been found in atopic dermatitis and psoriasis [51, 55, 56], indicating impaired epidermal differentiation, which is also noted in HS. As an early differentiation marker [56, 57], we examined cytokeratin-10 in the epidermis. Cytokeratin-10 expression is higher in healthy skin compared to lesional HS skin, which we also found in the epidermis equivalents irrespective of the cultivation period. The results of Dmitriev et al. confirm our findings. In contrast, Kurokawa et al. showed intense suprabasal cytokeratin-10 staining in HS epidermis. Furthermore, they investigated the expression of cytokeratin-10 in different stages of draining sinus tracts by immunohistochemistry. In the cornified epithelium, they detected a stronger expression of cytokeratin-10, which decreased in the less cornified and highly inflamed epithelium [57, 58]. On the other hand, cytokeratin-10 staining was again elevated in acantotic epithelium [57]. These results suggest that the degree of disease progression and/or inflammation has an impact on keratinocyte differentiation and thus may lower the expression of cytokeratin-10 in HS compared to healthy skin. For the evaluation of epidermis models in terms of proliferation capable keratinocytes within the model and thus viable keratinocytes, we used Ki-67 staining. Furthermore, a comparison between foreskin- and HS-epidermis models and the matching biopsies reveals whether the models correspond to the *in vivo* situation. In this regard, quantification demonstrated the trend of enhanced of Ki-67 positive cells in HS-epidermis models compared to foreskin-epidermis equivalents, which thus tended to correspond to the *in vivo* situation. By quantification, Dmitriev et al. also showed increased Ki-67(+) cells in HS skin in comparison with healthy control [43, 59]. The higher number of keratinocytes with the ability to proliferate seems to lead to psoriasiform hyperplasia in HS. Even though we could only demonstrate a trend toward higher numbers of Ki-67(+) cells in our quantification in HS-epidermis equivalents compared to foreskin-epidermis models, we can conclude from our data that the keratinocytes do not lose their ability to proliferate during cultivation.

Recognizing that HS is a chronic inflammatory skin disease, a research model should reflect the inflammatory patterns described in HS. It is known from the literature that the pro-inflammatory cytokines IL-1*β* and TNF-*α* are increased in lesional tissues, in the medium of cultured *ex vivo* explants, as well as in the blood of patients [9, 20, 60, 61]. Basic research indicates that IL-1*β* and TNF-*α* are key cytokines in HS pathogenesis [9, 60, 61]. Efforts have already been made to develop treatments for HS using antibodies that target specific cytokines. While two TNF-*α* antibodies are approved for HS treatment, no IL-1*β* antibody has yet received approval in clinical trials [62, 63]. Initial studies with anakinra showed promising effects [64, 65], but later case reports indicated no treatment success for severely affected HS patients [66, 67]. An open-label study on bermekimab demonstrated that it was well tolerated and effective [68]. However, a phase 2 study on bermekimab was discontinued early due to treatment failure [69]. Since the inflammatory pattern of HS is heterogeneous, it is likely that inhibiting just one cytokine will be insufficient. Even TNF-*α* antibodies are only effective in about 50% of HS patients [70, 71]. After a cultivation period of 14 days, our HS-epidermis models exhibit a trend of higher IL-1*β* and TNF-*α* secretions. The trend of IL-1*β* level reverses after 21 days of cultivation. In contrast, TNF-*α* release is below the detection limit in both HS- and foreskin-epidermis equivalents, suggesting that the cytokine profile may normalize after 21 days due to a lack of stimulation, and thus, the cultivation period of 21 days is too long. This hypothesis is further supported by the facts that Vossen et al. investigated the release of IL-1*β* and TNF-*α*, among others, after 24 hours of incubation in the medium of *ex vivo* explants from lesional HS tissue [29, 61]. Another explanation for this observation might be that the keratinocytes in HS are able to produce TNF-*α* [20], but TNF-*α* is mainly produced by cells of the aberrant infiltrate [20, 72], which is not present in our epidermis model.

## 5. Conclusions

In conclusion, primary adult keratinocytes from HS are able to form a multilayered epidermis model that expresses the essential differentiation markers similar to that of *in vivo* HS skin. In addition, HS-epidermal equivalents secrete the proinflammatory cytokines IL-1*β* and TNF-*α*. However, the HS epidermal models did not show these characteristics of native HS skin at a significant level. Our epidermis model is not intended to compete with *ex vivo* explants, but rather serves as the next step after monolayers, particularly for drug screenings. It is worth noting that the keratinocytes in the epidermis model are more like the natural environment than those in monolayers. However, to create an accurate model of human skin, it is necessary to include the dermis and subcutaneous tissues, along with the keratinocytes. This is often overlooked, even within *ex vivo* explants, as the subcutaneous tissue is commonly removed. In HS, immune cell infiltration is described as in psoriasis. Compensating for the absence of immune cells in skin models from psoriatic donors, Pouliot-Bérubé et al. added a proinflammatory cytokine cocktail during cultivation at the air-liquid interface. As a result, the cytokine addition enhanced the psoriatic phenotype in the *in vitro* cultured skin models [73]. Therefore, this is a possible approach to create a more distinctive phenotype in the *in vitro* HS epidermis models comparable to the native HS skin and should be evaluated in further studies.

## Data Availability

The data supporting the results of this study are available from the corresponding author upon reasonable request.
